# Improving the response to lenvatinib in partial responders using a Constrained-Disorder-Principle-based second-generation artificial intelligence-therapeutic regimen: a proof-of-concept open-labeled clinical trial

**DOI:** 10.3389/fonc.2024.1426426

**Published:** 2024-07-30

**Authors:** Tal Sigawi, Ram Gelman, Ofra Maimon, Amal Yossef, Nila Hemed, Samuel Agus, Marc Berg, Yaron Ilan, Aron Popovtzer

**Affiliations:** ^1^ Department of Medicine, Hadassah Medical Center, and Faculty of Medicine, Hebrew University, Jerusalem, Israel; ^2^ Sharett Institute of Oncology, Hebrew University, Hadassah Medical Center, Jerusalem, Israel; ^3^ Oberon Sciences, Kfar Tavor, Israel; ^4^ Area9, Copenhagen, Denmark; ^5^ Stanford University, Palo Alto, CA, United States

**Keywords:** thyroid cancer, salivary gland cancer, lenvatinib, artificial intelligence, drug-resistant cancer

## Abstract

**Introduction:**

The main obstacle in treating cancer patients is drug resistance. Lenvatinib treatment poses challenges due to loss of response and the common dose-limiting adverse events (AEs). The Constrained-disorder-principle (CDP)-based second-generation artificial intelligence (AI) systems introduce variability into treatment regimens and offer a potential strategy for enhancing treatment efficacy. This proof-of-concept clinical trial aimed to assess the impact of a personalized algorithm-controlled therapeutic regimen on lenvatinib effectiveness and tolerability.

**Methods:**

A 14-week open-label, non-randomized trial was conducted with five cancer patients receiving lenvatinib—an AI-assisted application tailored to a personalized therapeutic regimen for each patient, which the treating physician approved. The study assessed changes in tumor response through FDG-PET-CT and tumor markers and quality of life via the EORTC QLQ-THY34 questionnaire, AEs, and laboratory evaluations. The app monitored treatment adherence.

**Results:**

At 14 weeks of follow-up, the disease control rate (including the following outcomes: complete response, partial response, stable disease) was 80%. The FDG-PET-CT scan-based RECIST v1.1 and PERCIST criteria showed partial response in 40% of patients and stable disease in an additional 40% of patients. One patient experienced a progressing disease. Of the participants with thyroid cancer, 75% showed a reduction in thyroglobulin levels, and 60% of all the participants showed a decrease in neutrophil-to-lymphocyte ratio during treatment. Improvement in the median social support score among patients utilizing the system supports an ancillary benefit of the intervention. No grade 4 AEs or functional deteriorations were recorded.

**Summary:**

The results of this proof-of-concept open-labeled clinical trial suggest that the CDP-based second-generation AI system-generated personalized therapeutic recommendations may improve the response to lenvatinib with manageable AEs. Prospective controlled studies are needed to determine the efficacy of this approach.

## 1 Introduction

Drug resistance is the main obstacle to achieving a cure in patients with cancer (1). Multiple mechanisms are associated with developing resistance, which presents a major therapeutic challenge (2). The current methods for overcoming resistance are far from satisfactory (3).

Thyroid cancer is the fastest-increasing malignancy in the United States and the eighth most common cancer globally (4). The treatment of its most common form, differentiated thyroid cancer (DTC), consists of surgery, radiotherapy, and radioactive iodine (RAI) (5). Up to 15% of DTCs are refractory to RAI and carry a poor prognosis (5, 6). Lenvatinib, a multi-targeted tyrosine kinase inhibitor, was approved as a monotherapy for treating locally advanced or metastatic RAI refractory DTC (RR-DTC) (5). Lenvatinib is also approved for advanced renal cell carcinoma, endometrial carcinoma, and hepatocellular carcinoma (7). Despite its efficacy, lenvatinib is associated with the development of drug resistance and with a spectrum of adverse events (AEs), including hypertension, fatigue, proteinuria, and gastrointestinal disturbances, which often necessitate dose reduction, interruption, or permanent discontinuation (5, 6, 8–10). Traditional strategies to overcome resistance, such as combination therapies and dose escalation, have not been widely implemented into clinical practice for DTC treatment and may be accompanied by increased toxicity (10). Further, a temporary cessation of treatment (i.e., a “drug holiday”) carries the risk of cancer progression.

The Constrained Disorder Principle (CDP) defines complex systems by their degree of inherent variability (11). Integrating CDP-based second-generation artificial intelligence (AI) systems into treatment regimens has shown promising results in enhancing therapeutic outcomes, overcoming drug resistance, and minimizing AEs (12–17). Personalized variability-based regimens optimized drug effectiveness in patients with congestive heart failure, multiple sclerosis, and chronic pain (13–15, 17–20).

The present study aimed to assess for the first time the effect of personalized dynamic adjustment of lenvatinib dosages and administration timing, guided by an AI-driven mobile application, on drug efficacy and AEs in patients with drug resistance or intolerance.

## 2 Methods

### 2.1 Study design and ethical considerations

An open-labeled, prospective, single-center proof-of-concept clinical trial lasting 14 weeks was conducted to investigate the impact of an algorithm-based regimen on lenvatinib efficacy. Subjects were enrolled at the Hadassah-Hebrew University Medical Center in Jerusalem, Israel. The study adhered to the Declaration of Helsinki and Good Clinical Practice guidelines. The trial was registered at the Israeli Ministry of Health, No. MOH_2023–03-21_012425, NIH GOV No. NCT06321120.

### 2.2 Study population

Participants were eligible for enrollment if they were 18 or older, non-pregnant, had a pathologically confirmed malignancy treated with lenvatinib monotherapy, and suffered from partial or complete loss of response to treatment or dose-limiting AEs. Participants who could not provide written informed consent or did not possess a smartphone were excluded. Participants were recruited from the Hadassah-Hebrew University Medical Center Oncology Department in Jerusalem, Israel.

### 2.3 Second-generation AI system

Altus Care™ is a mobile application developed by Area9 Innovation Apps, aimed at facilitating the digitalization of treatment plans and research protocols (21, 22). Integrated with treatment algorithms utilizing second-generation AI, Altus Care™ enables randomized adjustments in medication dosages and administration times within predefined physician-approved ranges while also serving as a medication reminder for patients (14, 15, 18). This study utilized an algorithmic approach providing randomized dosing regimens of selected drugs (Oberon Sciences, Israel) (14, 15, 18). The application is regulatory-approved and fully complies with privacy laws.

### 2.4 Designing a personalized treatment plan

Dosages and administration times were tailored within individual predefined ranges to accommodate personalized therapeutic regimens. The first level of the algorithm, employed in the present study, utilizes a pseudo-random number generator to select dosages and administration times from the ranges stipulated by the physician (14, 15, 18, 23). Per protocol, the daily dose was limited to match or remain below the patients’ pre-enrollment dosage level. In the initial four weeks of the follow-up, participants followed a fixed standard regimen with the app as a reminder, allowing for an adaptation period. Subsequently, the algorithm-driven treatment plan was implemented for an additional ten weeks.

### 2.5 Follow-up parameters

#### 2.5.1 Tumor response to treatment

##### 2.5.1.1 (18)F-fluorodeoxyglucose positron emission tomography-computed tomography (PET-CT)

All Participants underwent PET-CT at the beginning and end of follow-up. Tumor response was assessed by the Response Evaluation Criteria in Solid Tumors version 1.1 (RECIST v1.1) (24) and PET Response Criteria in Solid Tumors (PERCIST) (25, 26) to determine complete response, partial response, stable disease, or progressing disease. The pre-enrollment PET-CT was considered as the baseline for comparison.

##### 2.5.1.2 Tumor markers

Participants with thyroid cancer were tested for thyroglobulin tumor marker levels at the beginning and end of the study.

##### 2.5.1.3 Neutrophil-to-lymphocyte ratio (NLR)

Complete blood count was tested at the beginning and end of the follow-up. NLR was proposed as a biomarker of disease progression and as a prognostication tool in patients with thyroid cancer treated with lenvatinib. Higher values indicate more severe disease and worse outcomes (27–29).

#### 2.5.2 Safety and adverse events monitoring

Safety assessments were performed throughout the study, including recording symptoms and emergency room visits or hospitalizations. It was achieved through monthly telephone check-ups, hospital and ambulatory medical records reviews, and the option for Participants to report AEs online via the application. Hematological and biochemical laboratory testings, urinalysis, and self-conducted home blood pressure monitoring were also executed throughout the study. AEs were assessed according to the National Cancer Institute Common Terminology Criteria for Adverse Events (CTCAE), version 5.0.

#### 2.5.3 Quality of life and functional status assessment

##### 2.5.3.1 EORTC QLQ-THY34

The European Organization for Research and Treatment of Cancer Quality of Life Thyroid Cancer Module (EORTC QLQ-THY34) is a validated quality-of-life (QoL) questionnaire for patients with thyroid cancer that was used for QoL assessment at the start and end of follow-up (30). It comprises 34 questions, classified into 16 symptomatic scales and one social support scale with an adjusted score of 0–100 for each scale. Higher scores mean a higher degree of symptoms or support (30).

##### 2.5.3.2 Eastern Cooperative Oncology Group (ECOG) performance status

ECOG status was assessed at the beginning and end of the follow-up. Patients were scored on a 0 to 4 scale ranging from fully-functional to completely-disabled, respectively.

### 2.6 Statistical analysis

Continuous variables are presented as median with minimum and maximum values, while categorical data are expressed as counts and percentages. Due to the small sample size, there was insufficient statistical power for inferential analysis.

## 3 Results

### 3.1 Patients’ baseline characteristics and general measures

Six Participants meeting the inclusion and exclusion criteria were enrolled, with one withdrawing consent shortly after enrollment. The final analysis included five Participants. **Table 1** presents the baseline demographic and clinical characteristics of the Participants. The median age was 73 (range 69–76), with two (40%) male patients. Four (80%) patients had thyroid carcinoma, and one had salivary gland carcinoma. All patients had metastatic or recurrent locally advanced disease at enrollment.

**Table 1:** Baseline characteristics.

Patient No.	1	2	3	4	5
Age (years)	76	69	70	73	76
Sex	F	F	M	F	M
Diagnosis	Follicular thyroid cancer	Poorly differentiated thyroid cancer	Mixed medullary-papillary thyroid cancer	Salivary adenoid cystic carcinoma	Papillary thyroid cancer
AJCC disease stage	III	IV-B	IV-B	IV-B	IV-B
Time since diagnosis (months)	36	3	35	216	1
Time since lenvatinib initiation (months)	20	1	7	21	1
Daily lenvatinib dose before enrollment (mg)	14	24	24	14	20
Comorbidities	HTN, dyslipidemia, polycythemia vera	HTN, S/P pulmonary embolism, hypothyroidism	HTN, dyslipidemia	HTN, dyslipidemia, T2DM	HTN, CKD, cardiac pacemaker
Metastases sites	Larynx, paratracheal	Liver, lungs, skeleton	Lungs	Lungs	Lungs
Previous treatments	Surgery, RAI, radiation	radiation, chemotherapy	Surgery, radiation	Surgery	none
ECOG performance status	1	2	0	0	1

AJCC, American Joint Committee on Cancer; ECOG, Eastern Cooperative Oncology Group; HTN, hypertension; T2DM, type-2-diabetes mellitus; CKD, chronic kidney disease; RAI, radioactive iodine.

### 3.2 The algorithm-based treatment regimen was associated with improving or stabilizing disease progression parameters

**Table 2** summarizes the patients’ engagement and results. Four (80%) patients completed the 14-week follow-up period. One patient (patient no. 2) completed a 12-week follow-up and dropped out due to treatment substitution under the impression of progressing disease. The median recommended daily dose of lenvatinib before study enrollment was 20 mg (range 14–24), and it was reduced to a median of 16.67 mg (range 12–24) according to the intended experimental treatment plan.

**Table 2:** Study results: measured parameters for each of the five patients before and after the intervention.

Parameter	Normal range	Patient no. 1	Patient no. 2	Patient no. 3	Patient no. 4	Patient no. 5
Follow-up duration (weeks)	–	14	12	14	14	14
Mean planned daily dose (mg/d)	–	14;12	24;19	24;24	14;12	20;16.67
Dose interruptions* (%)	–	24	9	19	15	26
Maximal interruption duration (days)	–	6	3	14	7	15
RECIST v1.1	–	PR	PD	SD	SD	PR
PERCIST	–	PMR	PD	SMD	SMD	PMR
Sodium (mmol/L)	135–145	140;138	135;136	137;141	139;139	137;140
ALT (U/L)	0–55	35;**159**	23;36	23;28	22;21	11;**166**
Creatinine (mg/dL)	0.5–1.2	0.87;1.08	0.46;0.44	0.8;0.7	0.91;0.97	1.6;1.6
Calcium (mg/dL)	8.4–10.2	9.3;8.9	9.1;9.1	8.3;9.4	11.2;10.3	10.8;10.2
Thyroglobulin (ng/mL)	0–55	44.8;**76**	**250;96.2**	0.74;0	irrelevant	**3058;305**
Hemoglobin (g/dL)	~12–16	14.2;13.6	10;11	14.7;13.9	15.8;15	13.9;**11.1**
Platelets (k/microL)	150–450	200;**130**	209;265	169;233	206;156	201;218
NLR	–	1.91;2.57	8.45;4.82	1.84;1	1.79;2.3	5.7;3.5
Proteinuria	–	Stable microalbuminuria	Negative	Negative	New microalbuminuria	Microalbuminuria; nephrotic-range proteinuria
Blood pressure	–	Stable	Added therapy	Stable	Stable	altered therapy
Reported AEs	–	None	Fatigue	None	None	Stomatitis, xeroderma
Grade 3 AEs	–	None	HTN	None	None	HTN, proteinuria
ECOG	–	1;1	2;2	0;0	0;0	1;1
ER visits	–	0	0	0	0	0

Results are displayed as a single value, text, or as (pre-intervention value, post-intervention value) for each parameter. Values that deviate significantly from the expected range or show notable changes are highlighted in bold.

*presented as a percentage of total treatment duration.

ALT, alanine aminotransferase; ECOG, eastern cooperative oncology group; ER, emergency room; HTN, hypertension; NLR, neutrophil-to-lymphocyte ratio; PD, progressing disease; PERCIST, PET response criteria in solid tumors; PMR, partial metabolic response; PR, partial response; RAI, radioactive iodine; RECIST, response evaluation criteria in solid tumors; SD, stable disease; SMD, stable metabolic disease.

**Figure 1:**
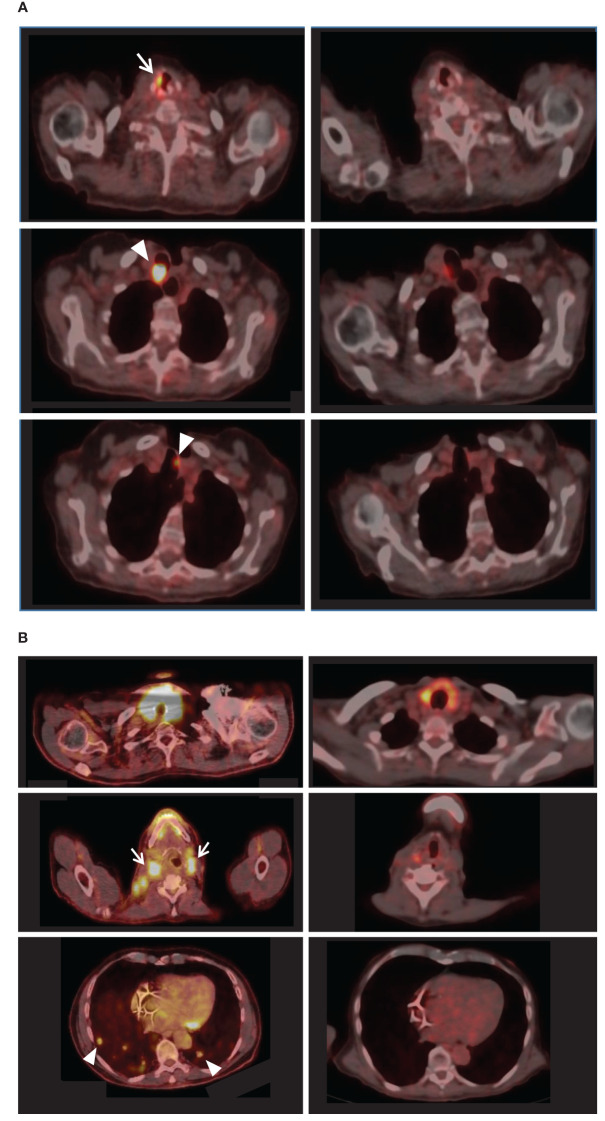
FDG-PET-CT results of the partial responders. For each patient, pre-intervention imaging results appear on the left, while comparable post-intervention sections appear on the right. **(A)** *Left:* Initially, patient no.1 exhibited right vocal cord uptake [white arrow] and hypermetabolic paratracheal lesions (maximal SUV 29.3) [arrowheads]. *Right:* Post-intervention, the lesions were almost entirely resolved (maximal SUV 5.8). **(B)** *Left*: Patient no.5 initially presented with extensive disease, including enlarged hypermetabolic thyroid, hypermetabolic cervical lymphadenopathy [e.g., white arrows], and multiple hypermetabolic lung metastases [e.g., arrowheads]. *Right*: Post-intervention PET-CT revealed reduced size and uptake of the thyroid, cervical lymph nodes, and lung metastases.

All patients completed an FDG-PET scan at the beginning and end of the study. According to the RECIST v1.1 and PERCIST criteria, 2 (40%) patients experienced a partial response, 2 (40%) had stable disease, and one (20%) showed progressing disease. **Figure 1** presents the imaging results of the partial responders. Notably, patient no. 1 exhibited a partial response despite having a recurrent disease during long-term lenvatinib treatment before enrollment. However, patient no. 2 demonstrated progressing disease, possibly due to a recently diagnosed widespread metastatic poorly differentiated carcinoma and a relatively poor performance status at enrollment.

**Figure 2:**
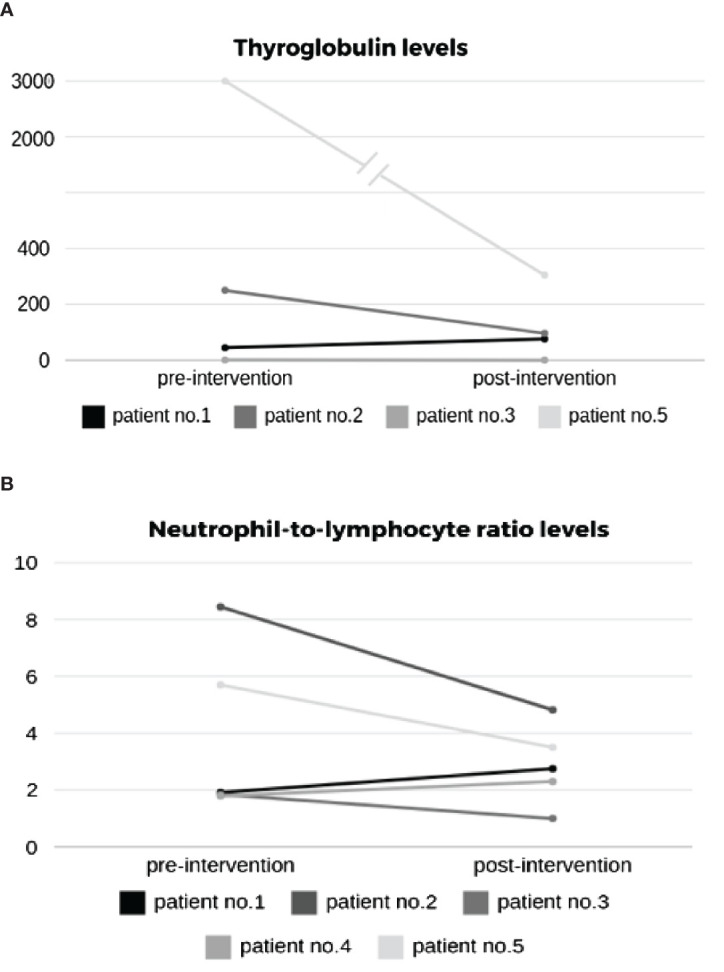
Effect of intervention on **(A)** thyroglobulin and **(B)** neutrophil-to-lymphocyte ratio (NLR) levels.

All four patients with thyroid cancer had thyroglobulin levels tested at the beginning and end of follow-up. Three (75%) patients showed a reduction in thyroglobulin levels by the end of follow-up (**Figure 2A**). The neutrophil-to-lymphocyte ratio (NLR) was calculated at the beginning and end of all patients’ follow-ups. Three (60%) patients showed a decrease in NLR during treatment. The remaining 2 showed mild elevation, with post-interventional values below 3 (**Figure 2B**).

### 3.3 The algorithm-based treatment regimen was associated with anticipated adverse events of lenvatinib

The documented AEs, classified by the CTCAE severity scale, include the following: *Grade 1*: ALT elevation (patients no. 1 and 5), thrombocytopenia (patient no. 1), anemia (patient no. 5), xeroderma (patient no.5), and proteinuria (patient no.4); *Grade 2*: Fatigue (patient no. 2) and stomatitis (patient no.5); *Grade 3*: Hypertension (patients no. 2 and 5) and proteinuria (patient no.5). No grade 4 AEs were identified. There were no emergency room visits or hospital admissions. A summary of the reported AEs and laboratory results is provided in **Table 2**.

### 3.4 The algorithm-based treatment regimen was associated with worsening symptom severity, improved social support, and stable performance status

EORTC QLQ-THY34 questionnaires were completed by 4 (80%) patients at the beginning and end of the follow-up period. The results showed an improvement in the median social support score and either stabilization or deterioration in 15 of the 16 median symptom scores (**Table 3**). These findings align with those from the phase IV EORTC QLQ-THY34 validation trial, which reported deterioration or stabilization across all tested scales in thyroid cancer patients treated with tyrosine kinase inhibitors within several months of starting therapy (30). The ECOG status, ranging from 0 to 2 at enrollment, remained unchanged throughout the follow-up period.

**Table 3:** Quality of life assessment by the EORTC QLQ-THY34 questionnaire.

Scale	Pre-intervention score	Post-intervention score	Difference
Exhaustion	44.4 (33.3, 66.7)	61.1 (33.3, 100)	16.7
Discomfort in the head and neck	44.4 (0, 66.7)	33.3 (22.2, 55.6)	-11.1
Voice	50 (33.3, 100)	55.6 (33.3, 100)	5.6
Hair problems	8.3 (0, 100)	33.3 (0, 100)	25
Swallowing	25 (0, 50)	25 (0, 33.3)	0
Dry mouth	16.7 (0, 66.7)	66.7 (0, 100)	50
Altered temperature tolerance	0 (0, 66.7)	33.3 (0, 100)	33.3
Body image	33.3 (0, 100)	66.7 (0, 100)	33.3
Rapid heartbeat	0 (0, 16.7)	16.7 (0, 66.7)	16.7
Shoulder functioning	0 (0, 66.7)	33.3 (0, 100)	33.3
Treatment- and disease-related worry	22.2 (0, 77.8)	38.9 (0, 88.9)	16.7
Joint pain	0 (0, 0)	16.7 (0, 33.3)	16.7
Tingling or numbness	0 (0, 16.7)	16.7 (0, 50)	16.7
Cramps	0 (0, 33.3)	16.7 (0, 66.7)	16.7
Worry about important others.	50 (0, 91.7)	62.5 (0, 91.7)	12.5
Impact on job or education	16.7 (0, 66.7)	50 (0, 66.7)	33.3
Social support	83.3 (0, 100)	94.4 (66.7, 100)	11.1

Sixteen symptomatic scales and one social support scale with an adjusted score of 0–100 for each scale are presented. Higher scores mean a higher degree of symptoms or support. Scores are presented as median (minimum value, maximum value).

### 3.5 Adherence to the treatment regimen

The patients demonstrated high engagement with the application. Dose interruptions, defined as temporary cessations in drug administration, were observed in all patients, whether planned (e.g., before elective medical procedures) or unplanned (usually to manage AEs). The interruptions occurred in a median of 19% (range 9–26) of the total lenvatinib treatment duration. The median time of the most extended single interruption for a patient was seven days (range 3–15).

## 4 Discussion

The data of this proof-of-concept feasibility clinical trial showed that it is possible to overcome resistance to lenvatinib using a CDP-based second-generation AI system. The data supports the safety of the system and its ability to improve clinical outcomes. The disease control rate, comprising complete response, partial response, and stable disease cases, was 80% within a 14-week follow-up. The FDG-PET scan-based RECIST v1.1 and PERCIST criteria showed partial response in 40% of patients (**Figure 1**) and stable disease in an additional 40% of the patients. One patient experienced a progressing disease. 75% of the patients with thyroid cancer showed a reduction in thyroglobulin levels, and 60% of patients showed a decrease in NLR during treatment (**Figure 2**). Improvement in the median social support score among patients utilizing the system supports an ancillary benefit of the intervention. No grade 4 AEs were recorded, no ER visits occurred, and no functional deterioration was noted.

Resistance to lenvatinib is a significant challenge that may cause loss of response to treatment through various molecular mechanisms related to cell death regulation, metabolism, histological transformation, and epigenetics (9, 10). Currently, methods are lacking to differentiate actual resistance from loss of response due to inadequate dosing, such as serum drug level monitoring. The digital pill, regulated by a CDP-based second-generation AI system, introduces an innovative approach to drug administration by incorporating variability signatures (15). Biological systems inherently display variability, which is mandatory for their proper function. This variability is governed by the CDP, which allows a degree of disorder within set boundaries (31–41). Deviating from this range may result in malfunction, disease, and possibly malignancies (42). Chronic medication administration triggers compensatory responses, diminishing effectiveness in up to half of patients with chronic illnesses, which may be associated with steady dosing (13). Research is advancing in using variability-based algorithms to counteract the compensatory responses to chronic medications (12, 21–23, 40, 42–56). These CDP-based AI systems personalize treatments by dynamically adjusting dosages and timing according to patient-specific and disease-specific variables, aiming to improve drug effectiveness (13–15, 18). Machine learning is also being explored to refine treatments by leveraging personalized variability, further enhancing chronic medication efficacy (12, 14, 21, 22, 42–62).

In the first level of this methodology, as utilized in the current study, lenvatinib dosages and administration times were randomized within the clinically approved ranges through a pseudo-random number generator, creating an open-loop system that functions independently of direct feedback (13–16). In the upcoming levels, the algorithm adapts treatments based on patient responses and clinical outcomes, evolving into a closed-loop system that personalizes therapy by learning from aggregated user data (15). Additionally, incorporating chronotherapy and biological variability markers represents a further refinement, aiming to align treatment regimens more closely with individual pathophysiology (15).

Lenvatinib gained approval for locally recurrent or metastatic progressive RR-DTC following the pivotal SELECT trial - a phase III, double-blind study that showed improved progression-free survival with lenvatinib versus placebo (63). Lenvatinib-related AEs occur in nearly all patients, with the majority being grade 3 or higher, especially in older patients (6, 64). In the SELECT trial, 88.7% of patients older than 65 years exhibited grade 3 or higher AEs, most commonly hypertension and proteinuria (64). Several retrospective analyses assessing the real-life treatment patterns, adherence, and AEs of lenvatinib in RR-DTC also showed a significant grade ≥3 AEs incidence (65–67). The absence of self-reported AEs by patients 1, 3, and 4 may be associated with alterations in the AEs perception after long-term pre-enrollment lenvatinib administration (68). Most guidelines advocate for an initial high dose of 24 mg/day of lenvatinib for thyroid cancer. However, the elderly and frail patients may struggle with compliance or may not be suitable candidates for high doses due to anticipated toxicities (5, 6, 64). In the SELECT trial, despite an initially planned dose of 24 mg/day, 67.8% of patients receiving lenvatinib required dose reductions due to AEs, resulting in a mean dose of 17.2 mg/day. Additionally, 82.4% experienced dose interruptions, and 14.2% discontinued treatment due to toxicity, with even higher rates noted in older patients (63, 64). Other studies have reported substantial dose reductions or interruptions exceeding 80% within a few weeks of treatment initiation (6). These interruptions may affect disease progression and outcomes, as more prolonged disruptions are associated with a shorter progression-free survival (69).

Retrospective studies have proposed planned drug holidays as a strategy to address intolerable lenvatinib AEs and prevent recurrent treatment interruptions and potential tumor regrowth (70, 71). However, planned drug holidays are not routinely implemented in clinical practice for RR-DTC treatment, primarily due to concerns regarding disease progression (72). Treatment interruptions are undesirable and can be caused by side effects. Interruptions may lead to loss of effect of the drugs.

Therefore, a method for reducing drug interruptions while maintaining sufficient dosing is needed. In our trial, patients experienced dose interruptions of varying frequencies and durations. Pauses in treatment that are randomly introduced are not the same duration as a typical “planned drug holiday.” One patient discontinued treatment due to disease progression, with no discontinuations attributed to AEs. We attribute reliable drug administration reporting via the application’s user-friendly online system to reflect real-life adherence to the treatment regimen.

Despite the limitations of being an open-label design and small sample size, this study introduces a novel treatment strategy for patients receiving lenvatinib. The study’s relatively short duration precludes assessment of the algorithm’s long-term impact. Additionally, using the app as a reminder can enhance patients’ adherence and outcomes, irrespective of the variability mechanism (20).

In conclusion, conventional fixed regimens fail to address the complexity of lenvatinib treatment, marked by a high incidence of adverse events, emerging resistance mechanisms, and limited alternative therapies. The CDP-based AI-assisted approach offers a user-friendly framework for developing and implementing innovative treatment strategies, benefiting non-responsive patients who cannot tolerate maximum doses. We advocate alternating dose reductions and administration times rather than interruptions or drug holidays. Further research is warranted to assess the impact of variability-based treatment regimens, integrated with AI and digital tools, in reducing dosing interruptions, managing adverse events, and enhancing tumor response. The system provides a novel option for overcoming drug resistance in patients with malignancy.

## Data availability statement

The raw data supporting the conclusions of this article will be made available by the authors, without undue reservation.

## Ethics statement

The studies involving humans were approved by Hebrew University Hadassah IRB. The studies were conducted in accordance with the local legislation and institutional requirements. The participants provided their written informed consent to participate in this study.

## Author contributions

YI: Conceptualization, Writing – original draft, Writing – review & editing. TS: Data curation, Investigation, Writing – original draft, Writing – review & editing. RG: Investigation, Writing – review & editing. OM: Conceptualization, Investigation, Writing – review & editing. AY: Data curation, Investigation, Writing – review & editing. NH: Data curation, Investigation, Writing – review & editing. SA: Data curation, Investigation, Writing – review & editing. MB: Conceptualization, Software, Writing – review & editing. AP: Conceptualization, Investigation, Writing – review & editing.
